# Braking Distance and Reaction Time Unchanged Across Hand-Use Conditions in Healthy Volunteers

**DOI:** 10.7759/cureus.99463

**Published:** 2025-12-17

**Authors:** Jacob Farley, Andrew Gaetano, Samuel Mircoff, Andrew Chen, Krishin Shivdasani, Dane Salazar, Nickolas Garbis

**Affiliations:** 1 Department of Orthopedic Surgery, Loyola University Chicago Stritch School of Medicine, Maywood, USA; 2 Department of Orthopedic Surgery and Rehabilitation, Loyola University Medical Center, Maywood, USA

**Keywords:** braking reaction time, driving simulator, hand dominance, kinematics, shoulder, upper extremity surgery

## Abstract

Purpose

The purpose of this study was to utilize driving simulator technology to evaluate braking time and distance, controlling for variables such as hand dominance and single versus both hand use.

Methods

This prospective observational study included healthy volunteers aged 18 and older with no recent upper extremity surgery. Data collected included age, hand dominance, and driving experience. Braking distance and reaction time were measured using Carnetsoft driving simulator software (Groningen, the Netherlands). The participants were instructed to use both hands, their dominant hand, or their non-dominant hand for each trial, accelerating to 60 miles per hour (mph) and decelerating to a stop, with 15 trials per participant, five per hand position. Linear mixed models were used to assess the association between hand use and braking time, accounting for multiple trials.

Results

Thirty-three healthy volunteers participated in this study. No statistically significant differences were observed in acceleration reaction time, brake reaction time, stop reaction time, or braking distance across the hand-use conditions, both hands, dominant hand, and non-dominant hand.

Conclusion

This study suggests that hand positioning does not significantly impact braking performance in healthy individuals under the conditions tested. This study highlights the potential use of driving simulators for evaluating specific driving abilities, which could inform rehabilitation and clinical assessments for individuals recovering from upper extremity surgeries, particularly as driver-assistive technologies evolve.

## Introduction

Driving simulator technology has emerged as a safe and effective tool for evaluating braking reaction time and workload in complex driving scenarios while minimizing safety concerns [1,2]. Commercial simulator platforms, including those produced by Carnetsoft, have been widely adopted in research due to their standardized driving environments and reliable data capture [3]. Applications of these technologies have extended into clinical research, including post-surgical biomechanics and surgical approach analysis [2]. Recently, simulator systems have been used to evaluate braking after total hip arthroplasty, assess neuromuscular activation after rotator cuff repair, and measure workload during functional driving tasks, yielding unique insights for functional evaluations in post-surgical populations [2,4-8].

Current guidelines for return to driving following upper extremity surgery vary widely. Recommendations include avoiding while casted or splinted, waiting six weeks or longer following shoulder arthroplasty, and waiting 2-7 weeks for operatively managed wrist fractures [9,10]. Badger et al. provided evidence that current recommendations are overly conservative and found that patients who underwent rotator cuff repair could safely return to driving at two weeks postoperatively [4]. Despite these general timelines, there is limited evidence-based guidance for determining individual readiness to resume driving.

Evaluating driving performance following shoulder or upper extremity surgery is of paramount importance to ensure the safety and well-being of both the patient and the general public. Most patients who have undergone a total joint arthroplasty base their decision to return to driving on direct surgeon advice [11-13]. Patients recovering from shoulder surgery often experience pain, reduced range of motion, and decreased strength, which may impair their ability to steer, merge, or react to hazards [14]. Identifying these limitations is crucial to ensure that patients are physically prepared to resume driving. Hasan et al. used driving simulator technology and found that patients who underwent arthroscopic surgery for rotator cuff or labral pathology recorded more collisions at six weeks postoperatively compared to controls [15]. They also found that surgery on the dominant driving arm resulted in greater collisions at six weeks than surgery on the non-dominant arm [15,16]. These results, when compared to current guidelines, implicate the need for further exploration and the potential revision of these guidelines. A pilot study using healthy volunteers can help establish the feasibility of using driving simulators to assess functional limitations, thereby informing future research in surgical populations.

The purpose of this study was to use driving simulation technology to evaluate braking distance and reaction time while controlling for hand dominance and hand use, both hands versus single-hand use. Testing individual components of driving burden can help determine where the risk lies in returning to safely operating a vehicle. We hypothesized that using both hands or the dominant hand would result in shorter braking reaction times and distances compared to using the non-dominant hand or a single hand. This study may help determine the feasibility of using a driving simulator for future prospective studies involving patients recovering from upper extremity surgery.

## Materials and methods

Study design and data collection

This was a prospective observational study conducted from July 2024 to November 2024 at a single academic institution under the supervision of two fellowship-trained orthopedic shoulder and elbow surgeons.

After approval from our institutional review board (Loyola University Chicago Health Science Division Institutional Review Board, with protocol ID 218509), the eligible participants were recruited from the medical student body and hospital faculty, including medical residents. Eligible volunteers included those who were age 18 years or older, possessed a valid driver’s license, and self-reported regular driving activity (greater than once per week). Exclusion criteria included any individuals with upper extremity injuries or surgery within six months or any lower extremity condition impairing pedal use. The participants were enrolled through convenience sampling and signed informed consent for participation in the study.

**Figure 1:**
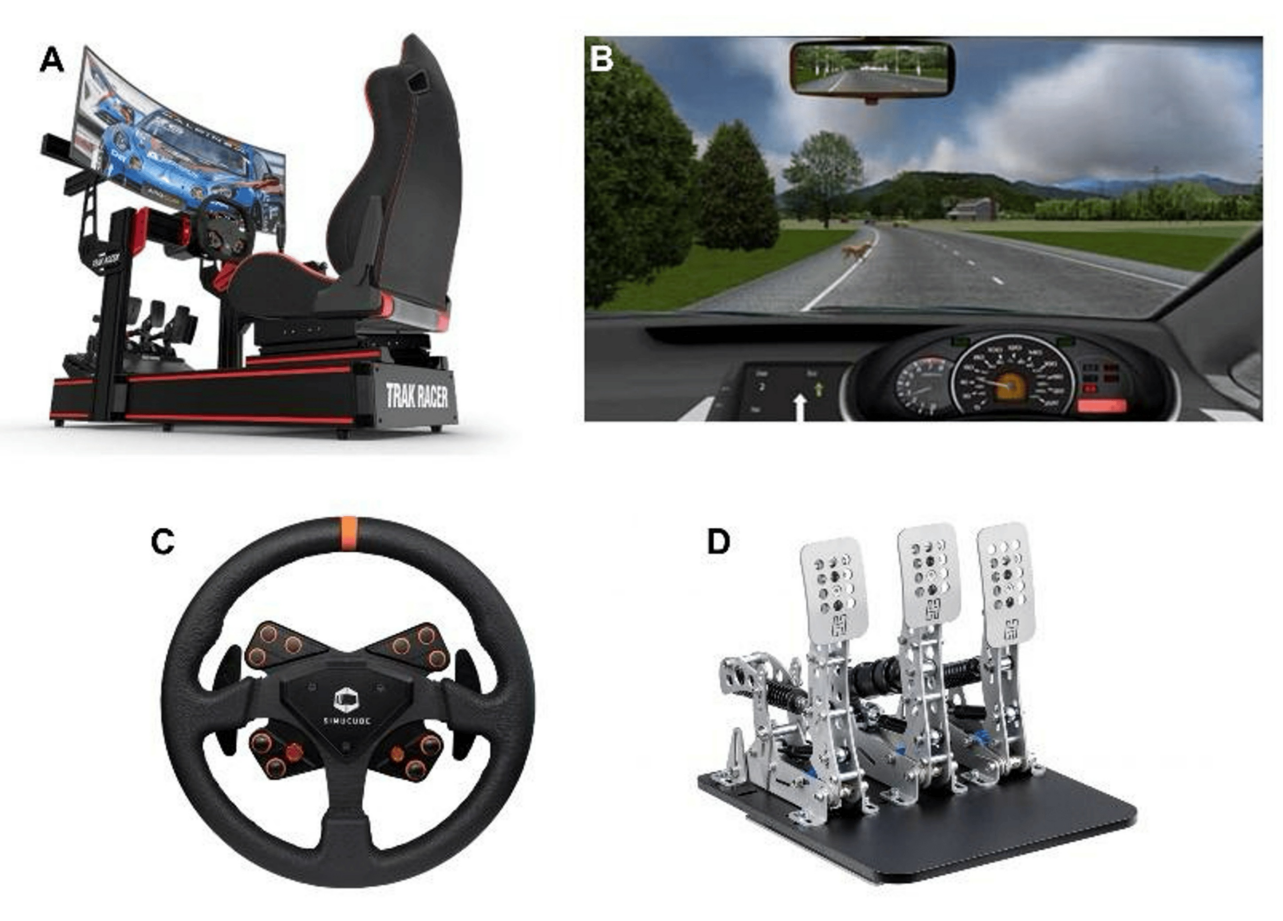
Driving simulator system. (A) Trak Racer TR160 driving simulator [17]. (B) Carnetsoft driving simulator screenshot [18]. (C) Simucube steering wheel [19]. (D) Heusinkveld pedals [20].

All participants performed 15 simulated driving trials with five trials conducted under each of three hand-use conditions: both hands, dominant hand only, and non-dominant hand only. The order of conditions was randomized for each participant to reduce order bias. During each trial, the participants were instructed to accelerate to 60 miles per hour (mph) and apply the brake pedal as quickly as possible upon receiving a standardized visual cue. The simulator environment consisted of a two-lane roadway with gentle curves, requiring minimal steering adjustments during braking. All driving trials were conducted using a Carnetsoft driving simulator system (Groningen, the Netherlands) (Figure 1). The simulator automatically recorded acceleration reaction time, brake reaction time, total stop time, and total braking distance.

Hardware consisted of a Trak Racer TR160 frame, Simucube steering wheel, and Heusinkveld pedals. The braking pedal in the simulator was configured with higher resistance than typical passenger vehicles, approximating a “panic-stop” force profile, which may influence braking effort. Calibration was performed before each participant using the manufacturer’s software to ensure full pedal travel and linearity.

All participants additionally completed a brief questionnaire, developed by the authors, in order to collect demographic variables and any potential confounding factors, including age, sex, hand dominance, driving experience, preferred transmission type, and motorsport experience (Table 1).

**Table 1:** Custom questionnaire developed by the authors to assess demographic variables and confounding factors.

Demographic and confounding factors questionnaire
1. Which hand do you consider to be your dominant hand?
2. How many years of driving experience do you have?
3. How many years of driving experience do you have with an automatic transmission?
4. How many years of driving experience do you have with a manual transmission?
5. Do you have motorsport experience?
6. If “yes” to question 5, how many years of motorsport experience do you have?

Statistical analysis

Descriptive statistics were performed with continuous variables reported as means and categorical variables reported as percentages. A priori power analysis indicated that a sample size of 32 participants would provide 80% power to detect a 5-meter difference in braking distance across hand-use conditions at an alpha level of 0.05. Linear mixed models were employed to assess the effect of hand use on braking performance outcomes (reaction time and braking distance), accounting for repeated measures within subjects. The Kenward-Roger approximation was applied to estimate adjusted confidence intervals and p-values used. All statistical analyses were conducted using R (version 4.4.3) (R Foundation for Statistical Computing, Vienna, Austria), with significance set at p < 0.05.

## Results

Participant demographics

All demographic information can be found in Table 2. A total of 33 healthy volunteers participated in this study. The average age of the participants at the time of obtaining their driver’s license and driving regularly is 16.206 and 16.382, respectively. Two of the participants currently drive manual transmission, and 14 have driven manual transmission in the past. One participant reported previous motorsport experience.

**Table 2:** Demographic Information.

Sample characteristics (n = 33)	n (%); mean
Sex	-
Male	25 (75.8)
Female	8 (24.2)
Age of receiving a driver’s license	16.206
Age of driving regularly	16.382
Currently drives a manual transmission	2 (6.1)
Have driven a manual transmission in the past	14 (42.4)
Motorsport experience	1 (3.0)

Reaction time and braking distance

No statistically significant differences were observed in acceleration reaction time, brake reaction time, stop reaction time, or braking distance across the three hand-use conditions: both hands, dominant hand, and non-dominant hand (Tables 3, 4). Acceleration reaction time showed consistent median values of approximately 0.45 seconds across all hand-use conditions. Brake reaction time medians were 0.71 seconds for both hands, 0.68 seconds for the dominant hand, and 0.72 seconds for the non-dominant hand. Stop reaction times were also similar across groups, with median values ranging from 5.38 to 5.69 seconds. Braking distance showed median values of 92.63 meters for both hands, 92.58 meters for the dominant hand, and 89.37 meters for the non-dominant hand.

**Table 3:** Median and interquartile range (Q1 and Q3) of reaction times and braking distance for different hand-use conditions.

Outcome measure	Both hands, median (Q1 and Q3)	Dominant, median (Q1 and Q3)	Non-dominant, median (Q1 and Q3)
Acceleration reaction time	0.45 (0.39 and 0.54)	0.45 (0.39 and 0.53)	0.45 (0.41 and 0.52)
Brake reaction time	0.71 (0.61 and 0.89)	0.68 (0.59 and 0.90)	0.72 (0.59 and 0.87)
Stop reaction time	5.69 (4.59 and 8.89)	5.38 (4.57 and 9.03)	5.38 (4.57 and 9.03)
Braking distance	92.63 (77.65 and 135.68)	92.58 (78.43 and 134.64)	89.37 (77.77 and 150.32)

**Table 4:** Pairwise differences in reaction times and braking distance between hand-use conditions with 95% confidence intervals (CI) and p-values.

Outcome measure	Difference (95% CI)	P-value
Acceleration reaction time		
Non-dominant versus dominant	-0.0016 (-0.02, 0.02)	0.9
Non-dominant versus both	-0.0076 (-0.03, 0.01)	0.4
Both versus dominant	0.0059 (-0.01, 0.02)	0.5
Brake reaction time		
Non-dominant versus dominant	-0.088 (-0.19, 0.02)	0.1
Non-dominant versus both	-0.036 (-0.14, 0.07)	0.5
Both versus dominant	-0.052 (-0.16, 0.05)	0.3
Stop reaction time		
Non-dominant versus dominant	-0.13 (-0.73, 0.48)	0.7
Non-dominant versus both	-0.05 (-0.66, 0.55)	0.8
Both versus dominant	-0.07 (-0.67, 0.53)	0.8
Braking distance		
Non-dominant versus dominant	-4.04 (-12.53, 4.44)	0.4
Non-dominant versus both	-1.63 (-10.1, 6.85)	0.7
Both versus dominant	-2.41 (-10.89, 6.07)	0.6

## Discussion

This study found no statistically significant difference in braking reaction time, stop reaction time, or braking distance across hand conditions in healthy individuals. These findings did not support our hypothesis that braking performance would be impaired with single-hand use or the use of the non-dominant hand. However, the study demonstrated that driving simulator technology is a safe tool for quantifying braking performance, offering consistent and reproducible metrics relevant to clinical assessments.

Previous studies have used simulator-based driving assessments to assess functionality following surgery. For instance, patients who have undergone hip arthroplasty have demonstrated significantly reduced braking force on simulators, with function typically normalizing around six weeks postoperatively [2]. Khaliq et al. conducted a systematic review on return-to-driving guidelines and noted a general recommendation of six weeks after shoulder arthroplasty [10]. However, these authors also highlighted the lack of high-quality, evidence-based studies to guide these recommendations. Our findings support the use of driving simulators as a research tool for studying functional driving capacity and guide future prospective trials in clinical populations, including patients recovering from upper extremity surgery.

Driving is a complex task that relies on strength, coordination, and rapid reaction time, all of which may be temporarily impaired following upper extremity surgery. Shoulder procedures in particular can cause pain, reduced range of motion, and reduced strength, which may hinder a patient’s ability to steer, merge, or brake effectively. In addition, patients in the early recovery period may be sleep-deprived, which can add another layer of complexity to analyzing driving safety. Resuming driving too early may also strain the healing joint, risking complications or delayed recovery. Simulator-based evaluations provide a safe, objective means for healthcare professionals to identify driving-related impairments, recommend appropriate modifications or restrictions, support optimal rehabilitation, and guide individualized return-to-driving decisions. In addition, vehicle technology such as adaptive cruise control and level II and III autonomous vehicles, partial automation and condition automation, respectively, may change the safety profile of postoperative driving.

Psychological factors also play an important role in driving readiness. Many patients experience fear, anxiety, and reduced confidence after surgery, which may impair their return to daily activities even when physically capable. Simulated assessments can address both physical and psychological barriers by helping healthcare professionals evaluate a patient’s mental readiness, provide appropriate support or counseling if needed, and ultimately restore confidence in performing daily activities, especially driving, fostering a safe and supported return to independence. From a legal and ethical standpoint, clinicians are responsible for guiding fitness to drive; simulator data can support these decisions, helping mitigate medicolegal risk while promoting public safety.

This study is novel in its use of driving simulation to isolate the effects of hand use and dominance on braking performance, a variable directly relevant to recovery from upper extremity surgery. Although our sample consisted of healthy individuals, the findings establish a baseline for functional expectations and demonstrate the feasibility of using simulation in clinical research. Future studies should incorporate surgical populations, assess longitudinal performance changes, and evaluate how simulator outcomes correlate with real-world driving behavior to better inform clinical guidelines.

This study has limitations. First, the braking system used in the simulator had higher resistance than most real-world vehicle brake pedals, which may have affected participants’ braking distances and reaction times. This was done with the intent of testing braking at the limit (i.e., panic stop). Second, the simulated driving environment included gently curved roads, requiring the participants to perform slight turning maneuvers with the steering wheel while simultaneously braking. The execution of this second task may impact reaction times and braking times [14]. Third, the sample size was relatively small despite meeting a priori power assumptions, which may limit the statistical power of the findings and the ability to detect subtle differences in braking force. Future research with larger and more demographically heterogenous cohorts might elucidate any differences that may have been undetected in our samples and further enhance the study’s generalizability. Future studies may additionally benefit from using a braking simulation with straight roads only, allowing for a more isolated and focused assessment of braking parameters. While this study does not replicate real-world driving conditions or include postoperative subjects, it establishes a foundational baseline for interpreting variability in braking performance related to hand use. This can assist in isolating specific risk factors that can impair postoperative driving ability.

## Conclusions

This study suggests that hand positioning does not significantly impact braking performance in healthy individuals under the conditions tested. Moving forward, these findings provide a framework for using driving simulators to evaluate functional driving abilities, which could inform rehabilitation and clinical decision-making for individuals recovering from upper extremity surgeries. As autonomous vehicle technology becomes more integrated into everyday life, the ongoing reassessment of safety considerations for postoperative patients will remain essential.
